# *Rickettsia parkeri* in Gulf Coast Ticks, Southeastern Virginia, USA

**DOI:** 10.3201/eid1705.101836

**Published:** 2011-05

**Authors:** Chelsea L. Wright, Robyn M. Nadolny, Ju Jiang, Allen L. Richards, Daniel E. Sonenshine, Holly D. Gaff, Wayne L. Hynes

**Affiliations:** Author affiliations: Old Dominion University, Norfolk, Virginia, USA (C.L. Wright, R. Nadolny, D.E. Sonenshine, H.D. Gaff, W.L. Hynes);; Naval Medical Research Center, Silver Spring, Maryland, USA (J. Jiang, A.L. Richards)

**Keywords:** *Rickettsia parkeri*, *Amblyomma maculatum*, Gulf Coast tick, bacteria, rickettsia, Virginia, vector-borne infections, dispatch

## Abstract

We report evidence that *Amblyomma maculatum* tick populations are well established in southeastern Virginia. We found that 43.1% of the adult Gulf Coast ticks collected in the summer of 2010 carried *Rickettsia parkeri*, suggesting that persons living in or visiting southeastern Virginia are at risk for infection with this pathogen.

*Rickettsia parkeri* is an obligate intracellular bacterium belonging to the spotted fever group of rickettsiae; this organism has recently been found to be pathogenic to humans (*1*). Infection with *R. parkeri* can be considered an emerging infectious disease, referred to as *R. parkeri* rickettsiosis, American Boutonneuse fever, and Tidewater spotted fever. Two confirmed cases of *R. parkeri* infections, including the index case in 2002, occurred in southeastern Virginia (*1*–*3*). Since then, 20 *R. parkeri* infections have been reported, mainly from the southern United States (*2*). In the United States, *Amblyomma maculatum* (family *Ixodidae*) ticks, commonly referred to as Gulf Coast ticks, are the only known natural vector of *R. parkeri*. *A. maculatum* ticks have been reported from 12 states: Alabama, Arkansas, Florida, Georgia, Kansas, Kentucky, Mississippi, Oklahoma, South Carolina, Tennessee, Texas (*1*,*4*,*5*), and Virginia (*6*). Sonenshine et al. reported finding individual *A. maculatum* ticks in Virginia in 1965 but concluded that populations had not become established (*7*).

We found large numbers of adult and some nymph *A. maculatum* ticks in Virginia. This population and the different life stages of the ticks indicate that they are now established in the state. Testing by real-time PCR and sequencing indicated that a high percentage of the ticks contained *R. parkeri* DNA.

## The Study

From May through September 2010, adult questing *A. maculatum* ticks were collected on flags at 3 locations in southeastern Virginia. Collection sites were selected to produce results that could be compared with those of previous surveys and to provide a comprehensive survey of southeastern Virginia (*8*). The first study site is 50 km inland and borders the Great Dismal Swamp in Chesapeake, Virginia. The second site, Back Bay National Wildlife Refuge, is <1 km from the Atlantic Ocean in Virginia Beach. The third site, in Portsmouth, borders the Elizabeth River.

The ticks were identified morphologically, and identity was confirmed as needed by molecular methods. DNA was extracted by using the DNeasy Blood and Tissue Kit (QIAGEN, Valencia, CA, USA) according to the manufacturer’s protocol and stored at –20°C until processing.

DNA samples were tested for *R. parkeri* DNA by real-time PCR with a MiniOpticon Real-Time PCR System (Bio-Rad, Hercules, CA, USA). Testing for *R. parkeri* DNA was by amplification and detection of a fragment of the *ompB* gene by using Rpa129F and Rpa224R primers and Rpa188 as the probe (Table 1). Samples negative for *R. parkeri* DNA were tested for *Rickettsia* spp. by amplifying a 111-bp fragment of the 17-kDa antigen gene (Table 1).

**Table 1:** Sequences of primers and probes used to test for *Rickettsia* spp. DNA in *Amblyomma maculatum* ticks collected from southeastern Virginia, April–September 2010*

Name	Sequence, 5′ → 3′	Gene	Fragment	Reference
Rpa129F	CAAATGTTGCAGTTCCTCTAAATG	*ompB*	96	J. Jiang et al., unpub. data
Rpa224R	AAAACAAACCGTTAAAACTACCG	*ompB*	96	J. Jiang et al., unpub. data
Rpa188Probe	6-FAM-CGCGAAATTAATACCCTTATGAGCAGCAGTCGCG-BHQ-1	*ompB*	96	J. Jiang et al., unpub. data
R17K128F2	GGGCGGTATGAAYAAACAAG	17-kDa antigen gene	111	J. Jiang et al., unpub. data
R17K238R	CCTACACCTACTCCVACAAG	17-kDa antigen gene	111	J. Jiang et al., unpub. data
R17K202TaqP	FAM-CCGAATTGAGAACCAAGTAATGC-TAMRA	17-kDa antigen gene	111	J. Jiang et al., unpub. data
190-FN1	AAGCAATACAACAAGGTC	*ompA*	540	(*1*)
190-RN1	TGACAGTTATTATACCTC	*ompA*	540	(*1*)
RompB11F	ACCATAGTAGCMAGTTTTGCAG	*ompB*	1895	(*9*)
RompB1902R	CCGTCATTTCCAATAACTAACTC	*ompB*	1895	(*9*)

*omp, outer membrane protein gene.

Three representative *A. maculatum* samples positive for *R. parkeri* by real-time PCR were confirmed by sequencing of a 540-bp fragment of the *ompA* gene. The fragments were amplified on an iCycler (Bio-Rad) by using primers 190-FN1 and 190-RN1 (Table 1). Samples positive for *Rickettsia* spp. but negative for *R. parkeri* had their *ompB* gene amplified and sequenced by using primers RompB11F and RompB1902R (Table 1). All PCR products for sequencing were purified by using Wizard PCR Preps DNA Purification System (Promega, Madison, WI, USA), and sequencing reactions were performed by using the BigDye Terminator v.3.1 Cycle Sequencing Kit (Applied Biosystems, Foster City, CA, USA) as described by the manufacturer and using appropriate primers (Table 1). Sequence similarities were identified by a BLAST search (http://blast.ncbi.nlm.nih.gov).

A total of 65 adult and 6 nymph *A. maculatum* ticks were collected (adults in May–September, nymphs in April). A total of 54 adults were collected from the Chesapeake site, 8 from the Virginia Beach site, and 3 from the Portsmouth site. Of the 6 nymphs collected, 5 were found feeding on a cotton rat at the Chesapeake site in April, and 1 was collected on a flag at the Virginia Beach site in September. Of the 65 total adult ticks tested, 29 (44.6%) were found by real-time PCR to contain *Rickettsia* spp. DNA, and 28 (43.1%) of the total adults collected contained *R. parkeri* DNA. Of the 6 nymphs collected, 4 were infected with *R. parkeri*; all were from the rat at the Chesapeake site. Of the *R. parkeri*–positive samples sequenced, maximum identity was seen with *R. parkeri* sequences (GenBank accession no. FJ986616.1). The rate of *R. parkeri*–infected ticks started out high in May (83% infected) and then decreased to no infected ticks in August (Table 2).

**Table 2:** Real-time PCR results for adult *Amblyomma maculatum* ticks collected from southeastern Virginia, USA, 2010

Month and collection site	Total no. ticks	No. (%) positive *for Rickettsia parkeri*
May		
Chesapeake	12	10 (83)
Virginia Beach	0	0
Portsmouth	0	0
Total	12	10 (83)
June		
Chesapeake	37	15 (40.5)
Virginia Beach	4	0
Portsmouth	0	0
Total	41	15 (36.5)
July		
Chesapeake	3	2 (66.7)
Virginia Beach	1	0
Portsmouth	3	1 (33.3)
Total	7	3 (42.9)
August		
Chesapeake	1	0
Virginia Beach	2	0
Portsmouth	0	0
Total	3	0
September		
Chesapeake	1	0
Virginia Beach	1	0
Portsmouth	0	0
Total	2	0
Total	65	28 (43.1)

Of the 3 *A. maculatum* ticks collected from the Portsmouth site, 1 was found by real-time PCR to be positive for *Rickettsia* spp. but negative for *R. parkeri*. Sequencing of a fragment of the *ompB* gene revealed this isolate to contain DNA with a 100% match to *Candidatus* Rickettsia andeanae isolate T163 (GenBank accession no. GU395297.1), a rickettsiae initially found in Peru (*9*).

## Conclusions

The discovery of such numbers and life stages of *A. maculatum* ticks in widely dispersed locations indicates that they are now established in southeastern Virginia. Finding adult *A. maculatum* ticks at the Portsmouth site was unexpected because this is the northernmost site at which we found these ticks and is a peninsula devoid of white-tailed deer, a major host for adult ticks (*10*,*11*).

That 43.1% of adult *A. maculatum* ticks collected from southeastern Virginia contained *R. parkeri* differs from reported rates of *R. parkeri* in *A. maculatum* ticks elsewhere in the United States. For *A. maculatum* ticks from Florida and Mississippi, *R. parkeri* infectivity rate is 28% (*2*); for ticks from Florida, Kentucky, Mississippi, and South Carolina, the average rate is 11.5% (*12*). For *A. maculatum* ticks collected from Georgia, an infectivity rate of 5%–11.5% has been reported (*13*). In Arkansas, only 3 of 207 *A. maculatum* ticks contained *R. parkeri* (*14*). Despite the high percentage of *R. parkeri* in the southeastern Virginia ticks, 27 of 28 positive samples came from 1 collection site. One explanation could be that *R. parkeri* is transovarially transmitted. Currently, there is no evidence that *R. parkeri* is transmitted transovarially by *A. maculatum* ticks, although transovarial transmission of *R. parkeri* has been shown in *A. americanum* ticks in the laboratory (*15*).

We also found an *A. maculatum* tick infected with *Candidatus* Rickettsia andeanae, which has rarely been reported in the United States (*2*). Whether *Candidatus* Rickettisa andeanae is pathogenic to humans is unknown, although it has been suspected to cause infections in persons in Peru (9).

Further research is needed to identify the vertebrate host(s) of *R. parkeri*. This information could be useful for controlling the transmission of *R. parkeri* to and from the vector, as well as predicting where *R. parkeri* may be present. Studies relating to transovarial transmission of *R. parkeri* in *A. maculatum* ticks would also be useful for predicting the spread of infections. Because *R. parkeri* is known to cause infection in humans, the presence of this pathogen in southeastern Virginia should be a health concern to persons in this region.
